# Update: Vitamin D_3_ and calcium carbonate supplementation for adolescents with HIV to reduce musculoskeletal morbidity and immunopathology (VITALITY trial): study protocol for a randomised placebo-controlled trial

**DOI:** 10.1186/s13063-024-08342-z

**Published:** 2024-07-22

**Authors:** Nyasha Veronica Dzavakwa, Molly Chisenga, Grace McHugh, Suzanne Filteau, Celia Louise Gregson, Lackson Kasonka, Katharina Kranzer, Hildah Banda Mabuda, Hilda Mujuru, Nicol Redzo, Sarah Rowland-Jones, Ulrich E. Schaible, Victoria Simms, Rashida Abbas Ferrand, the VITALITY team, Dan Hameiri-Bowen, Esther Gea-Mallorqui, Matthias Hauptmann, Cynthia Kahari, Christoph Leschczyk, Tafadzwa Madanhire, Tadious Manyanga, Kudakwashe Mutasa, Sandra Rukobo, Ruramayi Rukuni, Tsitsi S. Mudzingwa, Veronica Sunkutu, Mizinga Tembo, Cassandra Namukonda, Paul Kelly, Adrian Martineau, Kate Ward, Moherndran Archary, John Pettifor, Grace John-Stewart, Adeodata Kekitiinwa, Carl Lombard, Helen A. Weiss

**Affiliations:** 1 Biomedical Research and Training Institute, 10 Seagrave Road, Harare, Zimbabwe; 2 London School of Hygiene & Tropical Medicine, London, UK; 3 University Teaching Hospital, Lusaka, Zambia; 4 Musculoskeletal Research Unit, Bristol Medical School, University of Bristol, Bristol, UK; 5 Division of Infectious and Tropical Medicine, Medical Centre of the University of Munich, Munich, Germany; 6 Department of Paediatrics, University of Zimbabwe, Harare, Zimbabwe; 7 Nuffield Department of Medicine, University of Oxford, Oxford, UK; 8 Research Centre Borstel, Leibniz Lung Centre, Borstel, Germany; 9 MRC International Statistics and Epidemiology Group, London School of Hygiene & Tropical Medicine, London, UK

**Keywords:** Vitamin D_3_, Calcium carbonate, HIV, Bone density, Stunting, Pubertal delay, Adolescents, Immune function, Gut microbiome

## Abstract

**Background:**

Of the 2 million children living with HIV globally, 90% live in sub-Saharan Africa. Despite antiretroviral therapy, longstanding HIV infection is associated with several chronic complications in children including growth failure, particularly stunting and delayed puberty. Vitamin D deficiency, which is highly prevalent among children living with HIV in sub-Saharan Africa, has further adverse impact on bone health. This trial aims to establish whether supplementation with vitamin D_3_ and calcium carbonate improves musculoskeletal health among peripubertal children living with HIV. This paper is an update to an already existing protocol that was previously published in *Trials* in 2022 and details changes in the trial outcomes.

**Methods/design:**

We will conduct an individually randomised, double-blinded, placebo-controlled trial of weekly high-dose vitamin D_3_ (20,000 IU) plus daily calcium carbonate (500 mg) supplementation for 48 weeks. Eight hundred and forty children living with HIV aged 11–19 years taking ART for ≥ 6 months will be enrolled and followed up for 96 weeks. The primary outcome is DXA-measured total body less-head bone mineral density Z-score (TBLH-BMD) at 48 weeks and is an update to the previous primary outcome total body less-head bone mineral content adjusted for lean mass (TBLH-BMC^LBM^) Z-score. The primary outcome was updated to address the substantial differences in distributions of TBLH-BMC^LBM^ Z-score between the two sites as a result of software differences of the DXA machines. Secondary outcomes are DXA-measured TBLH-BMD Z-score adjusted for height at 48 weeks a new secondary outcome, lumbar spine bone mineral apparent density Z-score, number of respiratory infections, lean muscle mass and grip-strength at 48 and 96 weeks, and TBLH-BMD Z-score at 96 weeks. Sub-studies will investigate the effect of the intervention on vitamin D_3_ pathway metabolites and markers of bone turnover, intestinal microbiota, and innate and acquired immune function.

**Discussion:**

This is the largest trial to date of vitamin D supplementation in children living with HIV. Intervening to address deficits in bone accrual through childhood is critical for optimising adolescent and early adult bone health, and prevention of later adult osteoporotic fractures. Trial results will draw attention to the need to screen for and treat long-term comorbidities in children living with HIV in resource-limited settings.

**Trial registration:**

Pan African Clinical Trials Registry PACTR20200989766029. Registered on September 3, 2020. URL of trial registry record: https://pactr.samrc.ac.za

**Trial status:**

Participant follow-up completed; data analysis ongoing.

**Supplementary Information:**

The online version contains supplementary material available at 10.1186/s13063-024-08342-z.

## Introduction

### Background and rationale {6a}

**Fig. 1:**
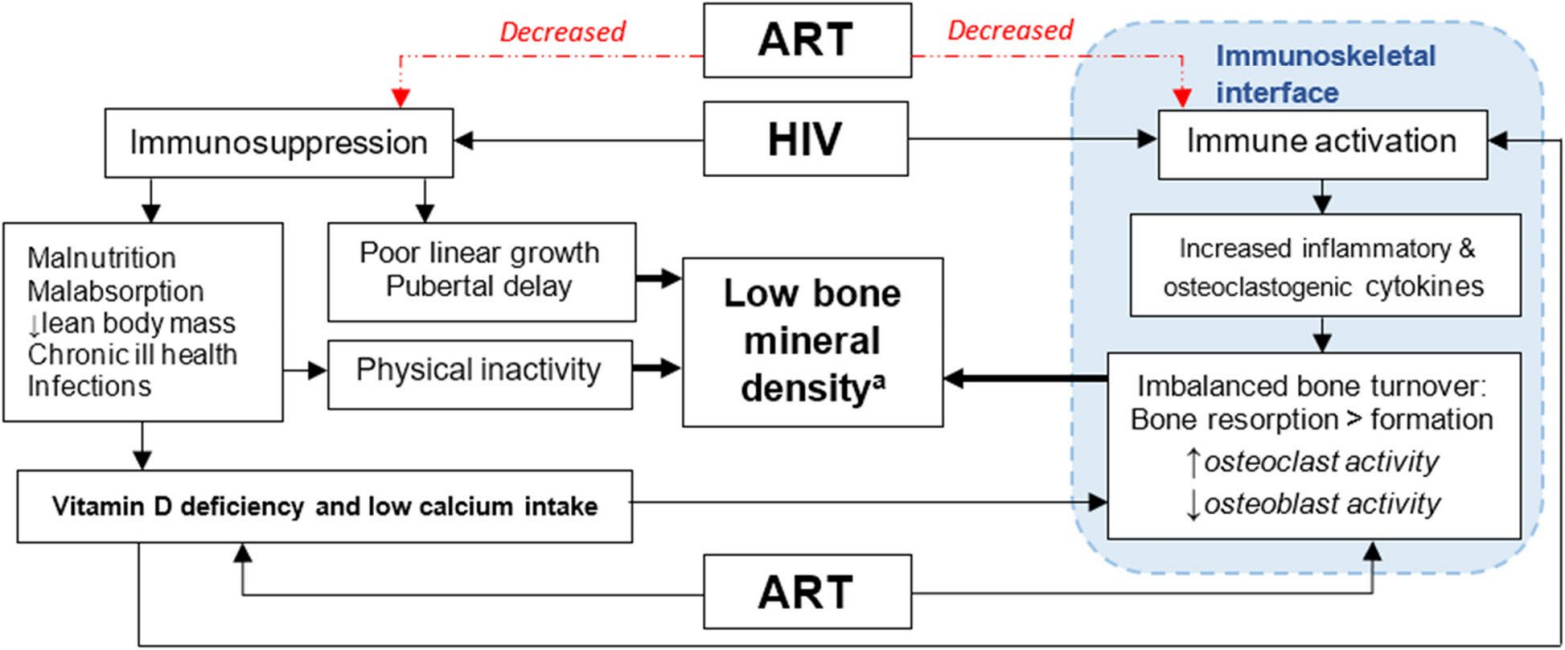
Immuno-pathogenesis of and risk factors for low bone density in children with HIV

The scale up of paediatric antiretroviral therapy (ART) programmes has resulted in increasing numbers of children surviving to adolescence and beyond [1, 2]. However, there is increasing recognition that HIV infection in children is associated with multisystem comorbidities despite ART [2]. HIV infection in children has substantial adverse effects on growth and musculoskeletal development, manifesting as stunting, pubertal delay, low bone density, and decreased muscle mass and function [1, 3]. Notably, these may be more profound among children living with HIV (CLWH) in sub-Saharan Africa than those in high-income settings, likely due to higher background rates of malnutrition, intercurrent infection, and delays in initiation of ART [2, 4]. Factors prevalent among CLWH that can compromise bone density include low muscle mass and strength, poor nutrition, inadequate dietary calcium, and vitamin D deficiency. HIV promotes dysregulated systemic immune activation which leads to an imbalance in osteoblastic/osteoclastic activity resulting in increased bone resorption relative to formation [1, 5–7]. Furthermore, ART drugs including both efavirenz and tenofovir (the first line ART in sub-Saharan Africa) may cause accelerated bone loss (Fig. 1) [8].

Puberty is a critical period for bone mass accrual; after cessation of linear growth and skeletal maturation, peak bone mass (PBM) is reached [8, 9]. PBM, which determines adult bone mineral density (BMD), accounts for 60% of lifetime osteoporosis risk [10], with a 10% decrease in PBM doubling adult fracture risk [11, 12]. Therefore, intervening to address deficits in bone accrual in childhood is important for both optimising current bone health and for future prevention of osteoporotic fractures. The “growth spurt” that occurs during adolescence offers a window of opportunity to optimise musculoskeletal health and minimise the chronic deleterious effects of HIV infection.

Vitamin D is the major regulator of calcium homeostasis and promotes intestinal calcium absorption and renal calcium reabsorption facilitating skeletal mineralisation. Vitamin D deficiency also dysregulates immune function and exacerbates HIV-mediated immune activation (implicated in the pathogenesis of comorbidities) and is associated with increased all-cause mortality in HIV infection [13, 14]. Furthermore, there is increasing recognition that the gut microbiome influences both immune and bone homeostasis [15, 16]. Small studies from high-income countries have shown that vitamin D supplementation improves bone density and muscle power among CLWH [17, 18]. These trials are promising but small and none has been conducted in sub-Saharan Africa, where 90% of CLWH live. Vitamin D deficiency is also a possible modifiable factor to reduce immune activation and high-dose vitamin D supplementation attenuated T-cell activation and monocyte activation [19, 20]. A recent meta-analysis demonstrated that vitamin D supplementation reduced the risk of acute respiratory infections (ARIs), although the risk reduction was modest [21, 22]. Vitamin D deficiency is associated with colonisation by proinflammatory bacterial species, and vitamin D supplementation may partially mediate its effects on bone through its impact on the gut microbiome [23].

This paper is an update to the 2022 VITALITY protocol [24] that was published in *Trials* reflecting changes made to the trial’s primary outcome. The primary outcome was updated to address issues with the substantial differences in the Z-score distributions of the original outcome total body less-head bone mineral content adjusted for lean mass Z-score (TBLH-BMCLBM) between Zimbabwean and Zambian participants. This was caused by the software differences in DXA machines being used to measure bone mass in the two countries, Hologic DXA in Zambia and Lunar iDXA in Zimbabwe. A more suitable alternative outcome total body less-head bone mineral density (TBLH-BMD) Z-score that is not affected by software differences was therefore identified.

### Objectives {7}

We hypothesise that adjuvant treatment with weekly vitamin D_3_ and daily calcium carbonate given during adolescence will promote bone accrual and mineralisation and maximise PBM, which will ultimately reduce the risk of adolescence and adulthood fractures among individuals living with HIV. Supplementation may also have beneficial impacts on muscle strength, immune function, and the gut microbiome. The trial objectives are to investigate the impact of adjuvant treatment with vitamin D_3_ and calcium carbonate given to CLWH taking ART for 48 weeks on:

1. Bone density assessed through dual-energy X-ray absorptiometry (DXA)
2. Muscle mass and strength
3. Risk of respiratory tract infections (RTI)

Outcomes will be determined at 48 weeks and participants will be followed for a further 48 weeks to investigate sustainability of intervention effect. Sub-studies embedded within the trial will investigate the effect of vitamin D_3_ and calcium carbonate supplementation on bone metabolism, body composition, immune function, and the gut microbiome.

## Trial design {8}

VITALITY is a phase III two-site individually randomised, double-blinded, placebo-controlled trial. It is a superiority trial with a 1:1 allocation ratio across two parallel groups.

## Methods: participants, interventions, and outcomes

### Study setting {9}

The trial will be conducted in Zambia and Zimbabwe. Participants will be recruited from outpatient HIV clinics at the Women and New-born Hospital at the University Teaching Hospital, Lusaka and at the Children’s Hospital at Sally Mugabe Central Hospital, Harare. These are the main public sector hospitals in the two cities and serve as teaching and referral hospitals.

### Eligibility criteria {10}

The inclusion criteria are:

1. Age 11–19 years with perinatally acquired HIV infection. Perinatally acquired HIV infection will be defined as the most likely source of HIV if the following case definitions are met: self-report of no sexual debut or blood transfusions, a history of orphan-hood due to maternal HIV disease and/or of a sibling death due to HIV, and characteristic clinical features (≥ 1 of stunting, history of recurrent minor infections, e.g. skin upper respiratory tract in childhood, and planar warts).
2. Taking ART for at least 6 months.
3. Having a firm home address and intending to remain there for 96 weeks.
4. Having a defined caregiver (those aged < 18 years).
5. Being aware of their HIV status (for those aged > 12 years).
6. Willing to give blood samples and rectal swabs.
7. Guardian consent and participant assent for those aged < 18 years; participant consent for those aged 18 to 19 years or emancipated minors.

The exclusion criteria are:

1. Any condition that may prove fatal during the study period (e.g. malignancy, end-stage HIV disease, or other conditions deemed likely fatal by the trial physician)
2. Taking TB treatment
3. Pregnant or breastfeeding
4. Having a condition likely to lead to lack of understanding of or cooperation with study procedures (e.g. neurocognitive disease, developmental delay, or psychiatric illness)
5. History of thyrotoxicosis, lymphoma, renal calculi, chronic renal disease, osteomalacia, hypercalcaemia, or a disorder of phosphate metabolism (all ascertained by participant report or records)
6. Physical (or radiological if available) signs of rickets or osteomalacia
7. Living in the same household as a trial participant (to avoid inadvertent mix-up of trial drugs)
8. High likelihood of non-adherence to trial medication (based on a persistent unsuppressed viral load and/or history of ART non-adherence not responsive to interventions as determined by trial physician)

### Who will take informed consent? {26a}

CLWH will be screened for eligibility to participate in this study using the above-mentioned criteria at HIV clinics. If they meet the inclusion criteria, the CLWH including their guardians (for those < 18 years) will be invited to the study clinics and given detailed information about the study by the study nurses. If they agree to participate, they will be screened for exclusion criteria by the study nurse and trial physician (if required) and sign the informed consent/assent as well as the guardian consent where required.

### Additional consent provisions for collection and use of participant data and biological specimens {26b}

Additional consent will be sought to biobank leftover blood samples for future studies. Specifically, consent will be sought for samples to be shipped to laboratories outside the country and for genetic testing of samples. A separate consent form will be used for this, with participants able to decline even if they consented to participation in the trial.

## Interventions

### Explanation for the choice of comparators {6b}

The control group will receive a placebo which is identical in appearance and taste. The use of placebo will facilitate blinding and controlling for the placebo effect.

### Intervention description {11a}

Eligible patients will be randomly assigned to receive either vitamin D_3_ and calcium carbonate or placebo. Vitamin D_3_ (cholecalciferol) will be given as a high-dose (20,000 IU) weekly dose (one tablet). Calcium carbonate will be given as a 500 mg daily dose (one chewable tablet). Placebo will look and taste identical to the investigational medicinal product (IMP). The intervention drugs and placebo were produced by KD CHEM Pharma B/h, Ujala Avenue, Narol-Sarkhej Road, Juhapura, Ahmedabad, Gujarat 380055, India.

### Criteria for discontinuing or modifying allocated interventions {11b}

Participants can leave the study at any time for any reason if they wish to do so without any consequences. Participation in this study can also be ended by the investigator if the participant is uncooperative and/or does not attend study visits. A participant will be considered to have withdrawn from the study if he/she does not attend for a study visit after a home visit (done within 1 week of a missed visit) or refuses to take the study drug on two or more occasions without there being a reasonable cause. Participant data that have been collected up to that point will be included in the analysis.

The study drug will be discontinued if participants experience a grade 4 adverse reaction (AR) or any suspected unexpected serious adverse reaction (SUSAR) based on an allergic reaction. See Table 1 for definitions of adverse reactions and events. Severity will be graded according to the modified DAIDS classification [25]. Discontinuation of the study drug will be considered in the event a participant experiences a grade 3 AR. Follow-up will continue as per protocol if the study drug is discontinued. If a participant becomes pregnant during the study, they will continue the study drug and be followed up (e.g. for grip strength, bone turnover markers, etc.), but will be excluded from DXA and peripheral quantitative computed tomography (pQCT) scanning given the small degree of radiation exposure associated with these investigations. The pregnancy will be monitored, and pregnancy outcome recorded. All participants enrolled into the study will have serum creatinine checked at week 12 and calcium and phosphate at weeks 12 and 24. Supplementation with calcium and vitamin D_3_ can rarely unmask subclinical primary hyperparathyroidism. If hypercalcaemia is detected at this stage, participation will be discontinued with onward referral to clinical services.

**Table 1:** Definitions of adverse reactions and events

	**Definition**
Adverse event (AE)	Any untoward medical occurrence in a patient or clinical trial subject to whom a medicinal product has been administered including occurrences that are not necessarily caused by or related to that product
Adverse reaction (AR)	Any untoward and unintended response to an investigational medicinal product related to any dose administered
Unexpected adverse reaction (UAR)	An adverse reaction, the nature or severity of which is not consistent with the information about the medicinal product in question set out in the Summary of Product Characteristics (SPC) or Investigator Brochure (IB) for that product
Serious adverse event (SAE) or serious adverse reaction (SAR) or suspected unexpected serious adverse reaction (SUSAR)	Respectively any adverse event, adverse reaction or unexpected adverse reaction that: • Results in death • Is life-threatening • Requires hospitalisation or prolongation of existing hospitalisation • Results in persistent or significant disability or incapacity • Consists of a congenital anomaly or birth defect • Is another important medical condition

### Strategies to improve adherence to interventions {11c}

Adherence will be encouraged through directly observed treatment (DOT) by a designated caregiver. At each visit, adherence will be monitored by counting remaining tablets and thorough check of the DOT diary that will be maintained by the caregiver. The DOT diary will be collected from all participants at completion of treatment at 48 weeks. In addition, a sub-study will assess adherence with an electronic medication monitoring device and correlate findings with self-report and with 25-hydroxy vitamin D_3_ (25(OH)D) levels at 48 weeks.

### Relevant concomitant care permitted or prohibited during the trial {11d}

ART and cotrimoxazole will be continued during the study. Drug history will be recorded at enrolment and all follow-up visits, including use of glucocorticoids. Any new drugs commenced during the course of the study will be discussed with the study physician to exclude the possibility of drug interactions. All participants will continue HIV care in their routine designated ART clinics, their HIV care being the responsibility of these clinics. Feedback of HIV viral load measurements, CD4 measurements, and any other relevant blood and/or radiology investigations will take place through the participant and their caregiver to the treating clinic. Participants enrolled into this study will not be prohibited from receiving any other relevant concomitant care.

### Provisions for ancillary care {30}

In the rare event that hypercalcaemia occurs, participants will be managed according to local guidelines with referral to the medical team on duty at each hospital site. The cost of management will be borne by the study. HIV viral load testing will be performed as part of the study and those who are found to have a viral load > 1000 copies/ml will receive enhanced adherence counselling and a repeat viral load 3 months later. Participants will be referred to their routine HIV care provider if their viral load remains elevated.

At each follow-up visit, participants will be asked about incident RTIs and how these events were managed (i.e. whether self-limiting, treated at a primary care clinic or in hospital) and their outcome. In addition, participants will be asked to contact the trial team and/or attend an unscheduled visit if they develop symptoms of RTIs at any point during follow-up. RTIs will be classified as either upper or lower respiratory tract infections and will be treated as per local guidelines by the study team including anti-microbial treatment where necessary.

The sponsor has insurance which will provide coverage for damage to research subjects through injury or death caused by any activities of the study. The insurance applies to the damage that becomes apparent during the study.

### Outcomes {12}

The primary is DXA-measured total body less-head (TBLH) bone mineral density (BMD) Z-score at 48 weeks. This outcome represents the gold-standard approach to size adjustment, crucial when assessing a growing skeleton. Size adjustment of DXA measures is important in children with chronic diseases such as HIV, where smaller size due to poorer growth and delayed puberty may explain findings of lower BMD. The mean TBLH-BMD Z-score will be compared between trial arms at 48 weeks after initiation of vitamin D_3_/calcium treatment.

This outcome is an update to the previous primary outcome total body less-head bone mineral content adjusted for lean mass (TBLH-BMC^LBM^) Z-score [24]. The primary outcome was updated because, during initial data cleaning and checking, it was noticed that distributions of the original primary outcome total body less-head bone mineral content adjusted for lean mass Z-score (TBLH-BMC^LBM^) differed substantially between Zimbabwean and Zambian participants. This was caused by the differences in DXA machines being used to measure bone mass in the two countries, Hologic in Zambia and Lunar iDXA in Zimbabwe. Thorough investigation identified the issue to have arisen from the version of the iDXA software. The reference population against which the Zimbabwean participant’s Z-score is derived was scanned with a comparatively older software version, making the Zimbabwean Z-score derivation sub-optimal, whereas this issue did not affect the Zambian Hologic software. TBLH-BMD Z-score was identified as the most suitable alternative outcome as it is not affected by software differences as the bone is measured in a slightly different way compared to TBLH-BMC.

The secondary outcomes are:

- TBLH-BMD Z-score adjusted for height at 48 weeks
- Height adjusted lumbar spine-bone mineral apparent density (LS-BMAD) Z-score at 48 weeks
- LS-BMAD and TBLH-BMD Z-scores at 96 weeks, to inform sustainability of intervention effects
- Lean muscle mass (kg) at 48 and 96 weeks
- Muscle strength (kg) at 48 and 96 weeks
- Number of RTIs at 48 and 96 weeks

All secondary outcomes will be assessed by comparing the mean between trial arms. All participants who have a DXA scan at 48 weeks will be included in final outcome analysis. The study will also assess the effect of the intervention on (i) vitamin D_3_ pathway metabolites, markers of bone turnover, and pQCT measured trabecular and cortical bone architecture; (ii) changes in fat and lean mass over time and the proportion of fat versus lean mass; (iii) intestinal microbiota by rectal swab analysis; and (iv) innate and acquired immune function and activation. Visits occurring at 4 weeks either side of the 48-week or 96-week outcome measurement point, i.e. between 44–52 weeks and 92–100 weeks, will be eligible for inclusion in analysis.

### Participant timeline {13}

See Table 2.

**Table 2:** Schedule of follow-up and investigations

	**Study period**										
	**Enrolment**	**Allocation**	**Post-allocation**								
	**0**	**0**	**2**	**12**	**24**	**36**	**48**	60*	**72**	84*	**96**
**Enrolment**											
Eligibility screen	**✓ **										
Pregnancy test (if post menarche)	**✓**										
Informed consent	**✓**										
Demographics, clinical and HIV history^a^	**✓**										
Current symptoms/adverse events	**✓**		**✓**	**✓**	**✓**	**✓**	**✓**	**✓**	**✓**	**✓**	**✓**
Dietary assessment and physical activity	**✓**						**✓**				**✓**
Allocation		**✓**									
**Intervention**											
Supply trial drug	**✓**		**✓**	**✓**	**✓**	**✓**					
Measurement of adherence to trial drug			**✓**	**✓**	**✓**	**✓**	**✓**				
**Assessments**											
Pubertal staging	**✓**						**✓**				**✓**
Anthropometry^b^	**✓**			**✓**	**✓**	**✓**	**✓**	**✓**	**✓**	**✓**	**✓**
Grip strength	**✓**						**✓**				**✓**
Standing long jump	**✓**						**✓**				**✓**
Bioelectric impedance (BIA) (Zambia only)	**✓**						**✓**				**✓**
***Radiological assessments***											
Total body and lumbar spine DXA scan	**✓**						**✓**				**✓**
pQCT scan (Zimbabwe only)	**✓**						**✓**				**✓**
iDXA imaging of hand and wrist (Zimbabwe only)	**✓**						**✓**				**✓**
***Laboratory assessments***											
Vitamin D pathway metabolites^c^ and iPTH	**✓**						**✓**				**✓**
Bone turnover markers^d^	**✓**						**✓**				**✓**
CD4 count and HIV viral load	**✓**						**✓**				**✓**
Serum calcium and phosphate levels				**✓**	**✓**						
Serum creatinine levels				**✓**							
Blood samples for immunology studies	**✓**						**✓**				**✓**
Full blood count (Zambia only)	**✓**										
Rectal swab for microbiome studies^e^	**✓**						**✓**				**✓**

^a^Age at diagnosis, WHO disease stage, nadir CD4 count, ART regimen, and duration of treatment; ^b^Standing and sitting height and weight (plus calf waist and hip circumference and skin fold thickness (triceps, subscapular, and suprailiac measurements in Zambia only)

^c^25OHD, 1,25(OH)_2_D, 24,25(OH)_2_D)

^d^P1NP, CTX

^e^first 120 participants recruited only

*brief visit to maximise retention

### Sample size {14}

A sample size of 838 participants has 80% power to detect a 0.21 effect size (standardised mean difference) for the TBLH-BMD Z-score difference between intervention and placebo arms (with 15% loss to follow-up). This sample size calculation was based on a previous version of the primary outcome that was later updated [24]. The effect size is based on the IMVASK study of 188 adolescents aged 11–17 living with HIV and 199 HIV negative controls of the same age. The difference in TBLH-BMC^LBM^ Z-score between groups increased with age [26]. We used a weighted analysis with 16-year-olds and 17-year-olds having a doubled weight, representing the 18–19 age group who were not recruited in the IMVASK study. In this dataset, the mean TBLH-BMC^LBM^ Z-score was − 0.84 in the HIV group and − 0.42 in the control group (a difference of − 0.42). We are aiming to detect an effect size half the size of this (0.21). The sample size has been rounded up to 840. An effect size of 0.21 TBLH-BMD Z-score approximates to a 20% reduction in fracture risk [27], making this a clinically appropriate, minimally important effect size to identify (Table 3) .

**Table 3:** Sample size with varying effect size, follow-up, and power

Minimum effect size (Z-score difference)	Follow-up	Power	Sample size per arm	Total sample size
0.21	85%	80%	419	838
0.21	85%	90%	562	1124
0.20	90%	80%	419	838
0.22	80%	80%	419	838

### Recruitment {15}

The VITALITY trial will use context-specific approaches to maximise participant recruitment in each country. In Zimbabwe, community mobilisation and sensitisation of potential participants will be done through collaborating with Community Adolescent Treatment Supporters (CATS) from Africaid-Zvandiri, an organisation that works with CLWH [28]. In addition, the CATS will distribute posters and fliers with information and contact details of the study to interested participants. In Zambia, mobilisation and sensitisation of potential participants will be done with the assistance of health care providers from four satellite clinics who will distribute posters and fliers to interested participants receiving care from the clinics. In both countries, responses to inquiries about participation in the study and any other study related queries are answered by the study team on a dedicated mobile telephone line at any time of the day. Each participant will be reimbursed for transport expenses incurred for study visits.

## Assignment of interventions: allocation

### Sequence generation {16a}

Eligible patients will be randomly assigned to receive either vitamin D_3_/calcium carbonate or placebo in a 1:1 ratio stratified by country, using block randomisation with variable block sizes of 2, 4, 6, 8, and 10 distributed in proportion to elements of Pascal’s triangle [29], using the ralloc command in Stata. Small block sizes are down weighted to prevent unmasking, and large sizes are down weighted to prevent treatment imbalance in the event enrolment is stopped. There will be no further stratification applied.

### Implementation {16c}

The randomisation schedule and allocation list will be generated using Stata, by an independent statistician based in London. The allocation list will be sent directly to the pharmaceutical company who will prepare and pack the study medication with pre-labelled trial participant identification numbers prior to participant enrolment.

### Concealment mechanism {16b}

The active drugs and placebo will be packed identically (1 blister pack of 4 in boxes of 13 for vitamin D_3_, 1 blister pack of 30 in boxes of 13 for calcium) and labelled with the trial participant ID. When a new participant is enrolled and given a new sequential ID number, the appropriate medication will be prescribed. Allocation is therefore concealed from all study staff as well as participants. The study ID number is pre-assigned to a trial arm before it is assigned to a participant.

## Assignment of interventions: blinding

### Who will be blinded {17a}

Participants and study personnel (including the site data managers and the trial statistician) and the pharmacist will be masked to treatment allocation.

### Procedure for unblinding if needed {17b}

All grade 4 SARs or SUSARs will be unmasked and discussed by the Trial Management Group (TMG) and the Data Safety and Monitoring Board (DSMB). Unmasking of grade 3 SARs will be considered individually by the TMG. The procedure is that the independent statistician will be asked to report trial arm to the TMG (in the case of a grade 4 adverse reaction) or directly to the study clinician if unmasked for other reasons.

## Data collection and management

### Plans for assessment and collection of outcomes {18a}

Research nurses and assistants in Zambia and Zimbabwe collect data at baseline and follow-up visits and record it on electronic clinical record forms (eCRFs) loaded onto tablets using Google Nexus™ tablets (Google, Mountain View, CA, USA) running OpenDataKit (ODK) software. In both countries, data will be extracted, processed, and saved to a Microsoft Access database. DXA data will be downloaded directly from the DXA software: Hologic DXA (to be used in Zambia) has an embedded Access database and iDXA (to be used in Zimbabwe) can export data as a.csv file. The European spine phantom will be used for cross calibration of the Hologic DXA and GE Lunar iDXA machines, in addition to the daily quality control scanning of the manufacturer provided phantom on each machine. Absolute values for BMC and bone area will be exported. pQCT and DXA will be repeated on 60 participants and grip strength will be repeated on 30 participants at their week 2 follow-up visit to assess reproducibility of the measurements. Data will be uploaded securely every 2 weeks to a server based at London School of Hygiene and Tropical Medicine (LSHTM). From there it will be downloaded to an encrypted and password-protected laptop, exported to Stata version 16.0, and merged into a single database for analysis. The participants will be identified by a unique trial specific number and/or code in all databases. Names will not be recorded in databases and any other identifying detail will be removed from all data files before analysis.

### Plans to promote participant retention and complete follow-up {18b}

Participants will receive a phone call reminder before study visits. Those who do not attend a visit will be followed up with phone calls and up to three home visits before being declared lost to follow-up. It will not be possible to collect primary outcome data for participants who move away or transfer out of the study.

### Data management {19}

Electronic clinical record forms (CRFs) will be used. All documents will be stored safely in confidential conditions and held for 5 years after the end of the trial in accordance with good clinical practice (GCP) principles. On all trial-specific documents, other than the signed consent forms and household locator form, the participant will be referred to by the trial participant number/code, not by name. The consent forms and locator forms will be kept separate from the CRFs to avoid linkage between participant name and the study ID. Direct access will be granted to authorised representatives from the sponsor, host institution, and the regulatory authorities to permit trial-related monitoring, audits, and inspections. In Zambia and Zimbabwe, clinical forms will be managed using electronic data management with eCRFs loaded onto tablets and incorporating predefined consistency checks. This process will be GCP compliant. To enable more efficient data management, the CRFs will be developed from a common template in both countries and use equivalent checks. Data from the laboratory forms will also be entered into eCRFs. Data will be uploaded daily from tablets to the cloud and stored in the LSHTM Central server. Further quality control checks will be performed weekly, including consistency cross-checks between forms, and queries will be raised with the data collection team. The trial Data Management Plan will be made available through LSHTM Data Compass.

### Confidentiality {27}

The participants’ data and specimens will be identified by a unique trial specific number and/or code in any database. Participants’ name and any other identifying details will be removed from all data files before analysis. The data team will sign a confidentiality agreement prohibiting disclosure of any patient-level information. The study databases will be password protected, and all electronic communications involving study data will be encrypted. Cleaned and locked datasets will have additional anonymisation (e.g. removal of exact birthdate) and be archived with a codebook in a curated repository accessible via catalogue search. Anonymised databases will be created and stored with all relevant data to facilitate any data transfer requests that are made subsequently. Ethical clearance from both Zambia and Zimbabwe will be sought before data are made available to third parties for further analysis.

### Plans for collection, laboratory evaluation, and storage of biological specimens for genetic or molecular analysis in this trial/future use {33}

Blood samples will be taken for immunological and bone mechanistic studies. Immunological studies undertaken will include adaptive immunity studies, whole blood sampling, and innate studies. The studies will be carried out in Lusaka, Harare, Oxford, and Borstel. In Zimbabwe, 10 ml of blood will be collected in EDTA tubes at 0, 48, and 96 weeks. In Zambia, 5 ml of blood will be collected in an EDTA tube and 10 ml in heparin tubes at 0, 48, and 96 weeks. Rectal swabs will be taken from the first 120 participants enrolled per site (with follow-up swabs in the same participants at stipulated timepoints). All samples will be stored at − 70 °C until assays are performed*.* DNA will be extracted from all baseline samples and plasma will be collected at all timepoints. Peripheral blood mononuclear cells (PBMCs) will be separated from blood samples (in Zambia only) and either freshly studied or cryopreserved for further studies. Innate immune cell phenotypes and activation markers as well as monocyte, macrophage, and neutrophil associated anti-microbial responses to standard stimuli including phagocytosis, respiratory burst, cyto- and chemokine responses will be analysed by flow cytometry and ELISA. Adaptive immune studies will include analysis of T-cell subsets, including regulatory T-cells, by flow cytometry and an assessment of T-cell activation using cell-surface activation markers. Additional studies of immune activation will be performed by measurement of soluble markers in plasma using multi-parameter bead array analysis. Rectal swabs will be analysed by comparative sequencing of 16S-rRNA amplicons (V3-V4 region) and principal component analysis (PCA) to identify significant changes in the microbiome composition by trial arm. Gut permeability will be monitored by detecting bacterial components such as endotoxin in plasma samples. Plasma markers for bone metabolism and DNA will be collected, frozen, and stored at − 80 °C. 25(OH)D, 1,25(OH)_2_D, and 24,25(OH)_2_D by tandem MS and intact parathyroid hormone (iPTH), N-terminal propeptide of type 1 procollagen (P1NP), and collagen type 1 cross-linked C-telopeptide (CTX) by electrochemiluminescence immunoassays will be measured at baseline, 48- and 96-weeks’ post-enrolment for plasma markers.

## Statistical methods

### Statistical methods for primary and secondary outcomes {20a}

The primary analysis will be modified intention-to-treat adjusting for trial site as a binary covariate. That is, participants will be analysed in the group to which they were randomised regardless of whether they received the assigned treatment. Participants who withdraw consent retrospectively for the use of their data will not contribute to analyses. Secondary analyses will include a per protocol analysis with a pre-specified adherence threshold that will be defined in the statistical analysis plan (Supplementary file 1). The mean TBLH-BMD Z-score at 48 weeks (primary outcome) and at 96 weeks after treatment will be compared between treatment groups, using linear regression to estimate the mean difference, and corresponding 95% confidence interval (CI). All analyses will adjust for country. We will also adjust for any of the following variables if they are imbalanced between trial arms at baseline: age, sex, and duration on ART. Between-group comparisons of binary outcomes will be analysed with logistic regression, to estimate odds ratios and 95% CI. Count data (e.g. number of adverse events, number of RTIs) will be analysed using Poisson regression. Continuous outcomes will be additionally adjusted for the baseline measure of the outcome to improve precision. For 96-week continuous outcomes, we will also include 48-week measures of the outcome using mixed-methods repeated measures.

### Interim analyses {21b}

No interim analysis is planned.

### Methods for additional analyses (e.g. subgroup analyses) {20b}

Pre-specified effect modification analyses will include site, sex, and baseline vitamin D level.

### Methods in analysis to handle protocol non-adherence and any statistical methods to handle missing data {20c}

Missing outcome data will be imputed using multiple imputation in Stata, according to plausible assumptions about missingness, if appropriate.

### Plans to give access to the full protocol, participant-level data, and statistical code {31c}

The full protocol, de-identified participant-level dataset, and statistical code for analysis of trial outcomes will be made publicly available in the LSHTM Data Compass repository, with searchable catalogued metadata. The dataset will be available to third party researchers 12 months after publication of the trial results, conditional on signing a data transfer agreement.

## Oversight and monitoring

### Composition of the coordinating centre and trial steering committee {5d}

The TMG will oversee the day-to-day management of the trial and ensure implementation of the VITALITY trial procedures according to protocol. The TMG will include the Chief Investigator, the trial coordinator, the site PIs and project managers, the project statistician, and the project laboratory studies lead. The TMG will meet monthly to review study progress and discuss trial implementation challenges. The main coordination centre of this study will be at the Biomedical Research and Training Institute, Harare, Zimbabwe.

A Trial Steering Committee (TSC) will provide oversight of the trial conduct and regularly review relevant information from other sources (e.g. other related trials). The TSC will consider recommendations of the DSMB and provide independent guidance and advice to the trial team. The TSC will comprise five external members (who are not involved with the study and are not affiliated to the institutions involved in this study). These will include experts in paediatric HIV medicine, musculoskeletal health, and immunology. The TSC will meet annually shortly after the annual DSMB meetings.

### Composition of the data monitoring committee, its role and reporting structure {21a}

A DSMB will be constituted to provide independent review of the study conduct and findings. The DSMB will comprise a chairperson with expertise in clinical trials who will be responsible for collating and communicating the views of the DSMB, an independent statistician, and two clinicians, at least one of them a paediatrician with research experience and expertise in the management of HIV infection. The DSMB will meet annually to review study approvals, progress, obstacles, and include statistics of enrolment, refusals to participate/non-enrolment, adverse events, withdrawals, and trial efficacy measures, by site. The DSMB will provide written feedback of their findings to the sponsor, the relevant research ethics committees, and the TSC.

### Adverse event reporting and harms {22}

Vitamin D toxicity is extremely rare. Excess vitamin D can cause hypercalcaemia. Symptoms of hypercalcaemia may include dehydration, vomiting, constipation (or less commonly diarrhoea) with abdominal pain, reduce appetite, irritability, fatigue, muscle weakness, and kidney stones. All participants will be closely monitored for signs and symptoms of hypercalcaemia and will be asked about side effects at each study visit and have safety bloods done (i.e. serum and calcium phosphate levels) at 12 and 24 weeks. All serious adverse events (SAEs) and grade 3 and 4 ARs including suspected unexpected serious adverse reactions (SUSARs) will be recorded on the relevant regulatory reporting forms and reported immediately to the site Principal Investigator who is responsible for ensuring the reporting of the event as per local ethical board guidelines to the relevant ethics committees, regulatory authorities, the DSMB, and the sponsor.

### Frequency and plans for auditing trial conduct {23}

The trial will be conducted in accordance with the principles of the Declaration of Helsinki and in full conformity with relevant regulations and with the 1996 ICH Guidelines for Good Clinical Practice (CPMP/ICH/135/95). The trial will be monitored by the LSHTM Research Governance and Integrity Office (RGIO) under their remit as sponsor and external trial monitors in Zimbabwe and Zambia to ensure adherence to good clinical practice.

### Plans for communicating important protocol amendments to relevant parties (e.g. trial participants, ethical committees) {25}

No change in the protocol will be implemented without prior review and approval of the regulatory authorities except where it may be necessary to eliminate an immediate hazard to the trial participants.

## Dissemination plans {31a}

A communication and dissemination strategy was developed and approved by all partners before the start of the trial. Stakeholder mapping was conducted, and communication channels and activities targeted at specific audiences were agreed. Dissemination will not be focused on the results of the trial only but will be a continuous activity throughout. This will ensure that any training materials, lessons learned, and interim results are shared in a timely manner. In addition, public engagement activities are planned at schools and/or adolescents clubs are planned in Zambia and Zimbabwe.

The following dissemination channels will be used throughout the trial: regular newsletter distributed by email, twitter (@TrialVitality), a dedicated website (www.vitality-trial.co.uk) and advertisement on partner and funder websites, meetings with national policymakers and implementors, presentations at local research institution, national conferences, and ministries of health. Results of the trial will be disseminated through presentations at national and international conferences, policy briefs and press releases, national dissemination meetings, dissemination meetings with health care workers at the clinics the trial is recruiting from, poster and leaflets designed to disseminate the trial to participants, and open access publications in peer-reviewed journals. The full anonymised dataset will be made available no longer than 12 months after publication of trial results through the LSHTM Data Compass curated repository.

## Discussion

The scale-up of ART programmes globally has resulted in a remarkable improvement in survival [1]. Thus, increasing numbers of children perinatally infected with HIV who would have died in early childhood in the pre-ART era who are now reaching adolescence [1, 2]. Growth failure, particularly stunting and delayed puberty, is one of the most common manifestations of paediatric HIV infection and adversely impacts skeletal development [4, 30]. While catch-up growth does occur following ART initiation, children may not reach their full growth potential especially if ART initiation is delayed. In sub-Saharan Africa, the median age of ART initiation over the past decade has been about 7 years compared to age 1 year in children with HIV in high-income settings [7, 10]. In addition, the use of tenofovir disoproxil fumarate, a component of first line ART, is associated with reduced bone density [10–12, 31].

Vitamin D deficiency is highly prevalent among children with HIV in sub-Saharan Africa and is a potential modifiable factor (combined with calcium supplementation given that diets in this region are low in calcium) for improving musculoskeletal health [17, 32, 33]. We hypothesise that supplementation with weekly vitamin D_3_ and daily calcium carbonate during puberty, a period of rapid growth, will promote bone accrual and mineralisation to ultimately optimise peak bone mass which is achieved at the end of puberty, and thus reduce the risk of childhood and adulthood fractures. Other potential benefits include improved muscle mass and strength, as well as innate and acquired immune function (given the immunomodulatory properties of vitamin D) and restoration of dysbiotic microbiota, which may consequently improve overall physical health and well-being.

The COVID-19 pandemic is likely to have an effect on implementation of this study. Lockdowns during epidemic waves may cause interruption in recruitment and the trial has also experienced delays in receipt of trial drugs. Procurement of consumables may be impeded by national and global stockouts. Shipment of consumables as well as samples may be delayed due to limited flights. The trial will implement strict infection prevention and control procedures including use of masks, sanitisation, and vaccination of research staff. SARS-CoV-2 testing will be available for research staff and participants if they experience symptoms.

### Trial status

The current protocol is version 4.0, dated 3 November 2022. Participant follow-up was completed in October 2023 and data analysis is currently ongoing until June 2025.

### Supplementary Information

Additional file 1. VITamin D for AdoLescents with HIV to reduce musculoskeletal morbidity and ImmunopaThologY Statistical Analysis Plan.

## Data Availability

Datasets generated during this study will be available, after analysis, in a public data repository. Before the datasets are made publicly available, all identifiable information will be removed. Investigators accessing data will be required to have ethical approval from the Zimbabwean and Zambian ethics committees.

## Abbreviations

ADP: Air displacement plethysmography
AR: Adverse reaction
ART: Antiretroviral therapy
ARIs: Acute respiratory infections
BIA: Bioelectrical impedance
BMAD: Bone mineral apparent density
BMC: Bone mineral content
BMD: Bone mineral density
CI: Confidence interval
CRF: Clinical record form
CTX: Collagen type 1 cross-linked C-telopeptide
CLWH: Children living with HIV
DSMB: Data Safety and Monitoring Board
DAIDS: Division of AIDS
DOT: Directly observed treatment
DXA: Dual-energy X-ray absorptiometry
eCRF: Electronic clinical record form
GCP: Good clinical practice
LS: Lumbar spine
LSHTM: London School of Hygiene and Tropical Medicine
ODK: OpenDataKit
PBM: Peak bone mass
PBMC: Peripheral blood mononuclear cell
PCA: Principal component analysis
P1NP: N-terminal propeptide of type 1 procollagen
pQCT: Peripheral quantitative computed tomography
PTH: Parathyroid hormone
RGIO: Research Governance and Integrity Office
SAEs: Serious adverse events
SARs: Serious adverse reactions
SUSAR: Suspected unexpected serious adverse reaction
TBLH: Total body less-head
TMG: Trial Management Group
TSC: Trial Steering Committee
