# Supplementary material for: Update: Vitamin D3 and calcium carbonate supplementation for adolescents with HIV to reduce musculoskeletal morbidity and immunopathology (VITALITY trial): study protocol for a randomised placebo-controlled trial
Source: Trials. 2024 Jul 22;25:499. doi: 10.1186/s13063-024-08342-z (PMC11264400; doi:10.1186/s13063-024-08342-z)
Supplement: Supplementary file 1 — Additional file 1. VITamin D for AdoLescents with HIV to reduce musculoskeletal morbidity and ImmunopaThologY Statistical Analysis Plan. [file 13063_2024_8342_MOESM1_ESM.docx]

**VITamin D for AdoLescents with HIV to reduce musculoskeletal
morbidity and ImmunopaThologY**


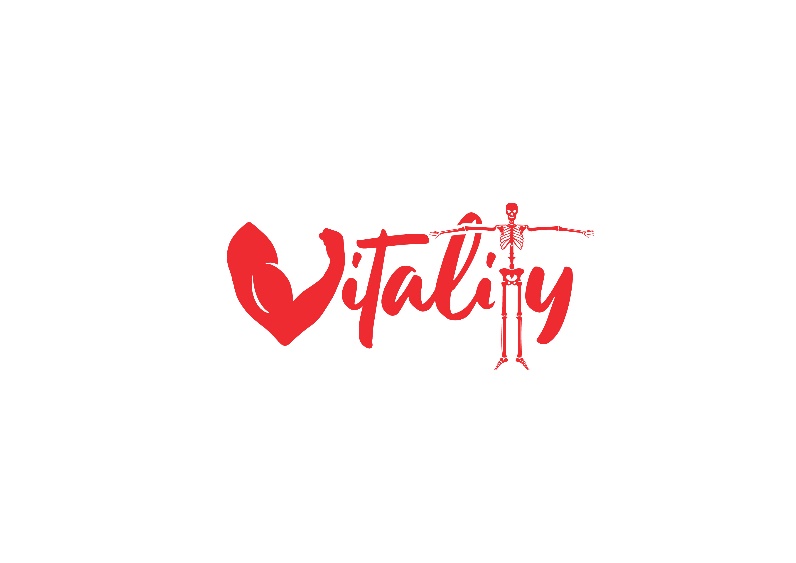


Version 1.1

Version 1.0 started 1 March 2022

PACTR reference: PACTR20200989766029

Date of registration: 3 September 2020

MRCZ reference: A/2626

Trial statistician: Dr Victoria Simms


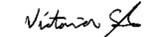


Signature .................................................... Date .....2 Nov 2023............

Principal investigator: Professor Rashida Ferrand


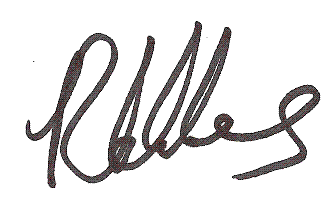


Signature..................................................... Date….2 Nov 2023………….

Data Safety and Monitoring Board Chair: Professor Grace John-Stewart


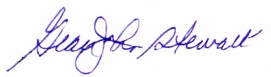


Signature..................................................... Date ..........2 Nov 2023................

## Amendments to SAP after signoff of version 1.0

| Date | SAP version | Protocol version and date | Reason for change |
| --- | --- | --- | --- |
|  | V1.0 | V1.3, 1 June 2021 | Initial version |
|  | V1.1 | V4.0, 3 November 2022 | Change of outcome |

# Contents

[Amendments to SAP after signoff of version 1.0 1](#_Toc115205928)

[Contents 2](#_Toc115205929)

[1. Investigators 4](#_Toc115205930)

[1.1. Contents of this SAP 4](#_Toc115205931)

[2. Description of the trial 5](#_Toc115205932)

[2.1. Background 5](#_Toc115205933)

[2.2. Objectives 5](#_Toc115205934)

[2.2.1. Primary objective 5](#_Toc115205935)

[2.2.2. Secondary objectives 5](#_Toc115205936)

[2.2.3. Bone mechanistic sub study objectives 6](#_Toc115205937)

[2.3. Trial design including blinding 6](#_Toc115205938)

[2.4. Eligibility screening 6](#_Toc115205939)

[2.4.1. Inclusion criteria 6](#_Toc115205940)

[2.4.2. Exclusion criteria 6](#_Toc115205941)

[2.5. Random allocation 7](#_Toc115205942)

[2.6. Duration of treatment 7](#_Toc115205943)

[2.7. Frequency and duration of follow-up 7](#_Toc115205944)

[2.8. Measures 7](#_Toc115205945)

[2.8.1. Baseline measures 7](#_Toc115205946)

[2.8.2. Co-primary outcome measures 7](#_Toc115205947)

[2.8.3. Secondary outcome measures 8](#_Toc115205948)

[2.8.4. Bone mechanistic sub study measures 8](#_Toc115205949)

[2.8.5. Moderators of treatment effects 9](#_Toc115205950)

[2.8.6. Safety measures 9](#_Toc115205951)

[2.9. Sample size considerations 9](#_Toc115205952)

[3. Data analysis plan – data description 10](#_Toc115205953)

[3.1. Recruitment and representativeness of participants 10](#_Toc115205954)

[3.2. Baseline comparability of randomised groups 10](#_Toc115205955)

[3.3. Completion of treatment and adherence 10](#_Toc115205956)

[3.4. Loss to follow-up and other missing data 10](#_Toc115205957)

[3.5. Adverse event reporting 11](#_Toc115205958)

[3.6. Assessment of repeatability of outcomes 11](#_Toc115205959)

[3.7. Descriptive statistics for outcome measures 11](#_Toc115205960)

[4. Data analysis plan – inferential analysis 11](#_Toc115205961)

[4.1. Analysis principles 11](#_Toc115205962)

[4.2. Main analysis of treatment difference 11](#_Toc115205963)

[4.2.1. Analysis of primary outcomes 11](#_Toc115205964)

[4.2.2. Analysis of secondary outcomes 12](#_Toc115205965)

[4.3. Statistical considerations 12](#_Toc115205966)

[4.3.1. TBLH-BMD and adjustment for height 12](#_Toc115205967)

[4.3.2. Time points 12](#_Toc115205968)

[4.3.3. Stratification 12](#_Toc115205969)

[4.3.4. Missing baseline data 13](#_Toc115205970)

[4.3.5. Missing outcome data 13](#_Toc115205971)

[4.3.6. Per protocol analysis 13](#_Toc115205972)

[4.3.7. Method for handling multiple comparisons 13](#_Toc115205973)

[4.4. Sensitivity analysis 13](#_Toc115205974)

[4.5. Planned subgroup analysis 13](#_Toc115205975)

[4.6. Interim analysis 14](#_Toc115205976)

[4.7. Process of unblinding 14](#_Toc115205977)

[5. Software 14](#_Toc115205978)

[6. Schedule of assessments and measures 14](#_Toc115205979)

[Figure 1: CONSORT Flow Diagram VITALITY trial 15](#_Toc115205980)

[Table 1: schedule of assessments 16](#_Toc115205981)

[Table 2: Baseline characteristics 17](#_Toc115205982)

[Table 3: Effect of the intervention 18](#_Toc115205983)

[Table 4: Effect of the intervention 18](#_Toc115205984)

[7. References 19](#_Toc115205985)

[Appendix 1: scoring 20](#_Toc115205986)

[Appendix 2: Peak height velocity 22](#_Toc115205987)

# Investigators

Principal investigator

Name: Rashida Ferrand

Address: Biomedical Research and Training Institute. 8 Ross Avenue, Harare, Zimbabwe

Email: [Rashida.ferrand@lshtm.ac.uk](mailto:Rashida.ferrand@lshtm.ac.uk)

Trial statistician

Name: Victoria Simms

Address: Biomedical Research and Training Institute. 8 Ross Avenue, Harare, Zimbabwe

Email: [Victoria.simms@lshtm.ac.uk](mailto:Victoria.simms@lshtm.ac.uk)

Data manager

Name: Nicol Redzo

Address: Biomedical Research and Training Institute. 8 Ross Avenue, Harare, Zimbabwe

Email: [nicol.redzo@gmail.com](mailto:nicol.redzo@gmail.com)

Study coordinator

Name: Nyasha Dzavakwa

Address: Biomedical Research and Training Institute. 8 Ross Avenue, Harare, Zimbabwe

Email: [Nyasha.dzavakwa@lshtm.ac.uk](mailto:Nyasha.dzavakwa@lshtm.ac.uk)

## Contents of this SAP

This SAP refers solely to the analysis of the primary and secondary trial outcomes (i.e. the analysis to be performed in producing the main results paper) and the bone mechanistic analysis. This SAP mentions data for microbiome and immunology studies but it does not describe the analysis associated with those variables.

# Description of the trial

## Background

The statistical analysis plan (SAP) for the VITamin D for AdoLescents with HIV to reduce musculoskeletal morbidity and ImmunopaThologY (VITALITY) trial is presented here. The plan defines *a priori* the analyses that will be completed for the primary and secondary outcomes of the study, including sub-group analysis. The plan adheres to CONSORT guidelines[1] , International Conference on Harmonisation requirements (ICH) and Good Clinical Practice (GCP) guidelines (E6(R1)).

Childhood HIV infection is associated with a heavy burden of comorbidities, which have received little focus. Growth failure, particularly stunting and delayed puberty, is one of the most common manifestations of HIV infection, and adversely impacts skeletal development.

The purpose of this trial is to establish whether supplementation with vitamin D_3_ and calcium carbonate improves musculoskeletal health among peripubertal children living with HIV (CLWH). Puberty is a period of rapid growth and musculoskeletal development, culminating in the achievement of peak bone mass (PBM), a key determinant of future adult fracture risk; similarly, peak muscle mass is a key determinant of age-related sarcopenia. Puberty is therefore the optimal period for providing this intervention as it is a period when there is rapid development of the skeleton with bone and muscle growth. It is anticipated that an intervention during this critical period of growth will improve bone accrual, conferring a reduction in future adult fracture risk. The intervention may have more immediate benefits: improving linear growth, muscle strength and immune function, and may subsequently improve overall physical and consequently social inclusion, enabling CLWH to participate more in schooling, sports and social activities. In addition, the trial will draw attention to the need to screen for and treat long-term comorbidities that occur in CLWH taking anti-retroviral therapy (ART).

The aim of this trial was to investigate the impact of weekly high-dose (20,000IU) vitamin D_3_ (cholecalciferol) plus daily 500mg calcium carbonate supplementation for 48 weeks on musculoskeletal health and immune regulation in children and adolescents living with HIV aged 11-19 years in Zambia and Zimbabwe. This SAP will be finalised prior to unblinding of treatment allocation.

## Objectives

### Primary objective

To investigate whether treatment with vitamin D_3_/calcium carbonate results in increased bone density in adolescents living with HIV on ART.

### Secondary objectives

To investigate the intervention effect of vitamin D_3_/calcium carbonate, in adolescents living with HIV on ART, on

- muscle mass for height squared (measured as fat-free mass)
- muscle strength (measured as grip strength)
- number of respiratory infections

### Bone mechanistic sub study objectives

To investigate the intervention effect of vitamin D_3_/calcium carbonate, in adolescents living with HIV on ART, on

- vitamin D_3_ pathway metabolites and markers of bone turnover
- tibial bone architecture measured by pQCT (Zimbabwe only)
- predicted tibial bone strength measured by pQCT (Zimbabwe only)
- lower limb muscle and sub-cutaneous fat volume measured by pQCT (Zimbabwe only)

## Trial design including blinding

The trial is individually randomised and placebo-controlled with allocation ratio 1:1. It is conducted at two sites, in Harare, Zimbabwe and Lusaka, Zambia. Study participants, care providers, outcome assessors and statisticians remain masked to treatment allocation per protocol. Care providers could be unmasked upon medical need of a participant. Weekly high-dose (20,000IU) vitamin D_3_ (cholecalciferol) plus daily 500mg calcium carbonate supplementation or placebo of identical appearance were dispensed by study pharmacists for 48 weeks. Participants attended a study visits two weeks after enrolment into the trial, and then at 12, 24, 36, 48, 72 and 96 weeks. All participants, study personnel and the trial statistician are blinded to treatment arm.

The trial started enrolment in February 2021 and completed enrolling 842 participants (from a target of 840) in November 2021. The last participant will complete the 48-week outcome in October 2022 and the 96-week outcome in September 2023.

## Eligibility screening

### Inclusion criteria

Eligibility for the trial is assessed at a screening visit.

1. Age 11-19 years
2. Perinatally-acquired HIV
3. Taking ART for at least 6 months
4. A firm home address accessible for visiting and intending to remain there for 96 weeks
5. Willing to agree to participate in the study and to give samples of blood and rectal swabs
6. Defined care-giver able to provide informed consent for child to participate in trial (for those aged below 18 years, unless emancipated minor)
7. HIV status disclosed to participant for those aged older than 12 years (13-19 years)

### Exclusion criteria

These exclusion criteria are assessed during the screening process.

1. Any condition (except HIV) that may prove fatal during the study period (e.g. malignancy, end-stage HIV disease or other conditions deemed likely fatal by the trial physician)
2. Taking TB treatment
3. Pregnant or breast-feeding
4. Condition likely to lead to lack of understanding of study procedures or to uncooperative behaviour e.g. neurocognitive disease, developmental delay or psychiatric illness
5. Living in the same household as a trial participant (to avoid inadvertent mix-up of trial drugs)

The following exclusion criteria are assessed after completion of consent. This is because they require the gathering of detailed medical information that requires informed consent.

1. History of thyrotoxicosis, kidney stones, lymphoma
2. History of chronic renal disease
3. History of hypercalcemia
4. History of a disorder of phosphate metabolism
5. Physical or radiological (if available) signs of rickets
6. History of osteomalacia

## Random allocation

Study IDs were pre-determined and were randomly allocated into intervention and trial arm by an independent statistician using block randomisation stratified by country, with block sizes of 2, 4,6, 8 and 10. The allocation list was sent directly to the pharmaceutical company which prepared, packaged and labelled the study medication. Random allocation occurs at the moment a new participant is assigned a study ID.

## Duration of treatment

The study drug is taken for 48 weeks. It is prescribed by a pharmacist at baseline, and 2-, 12-, 24- and 36-weeks post-enrolment.

## Frequency and duration of follow-up

Study visits take place at enrolment, and at 2, 12, 24, 36, 48-, 60-, 72- and 96-weeks post-enrolment. See the Schedule of Assessments for details.

The primary outcome will be measured 48 weeks post-enrolment, with a 4-week window (i.e. 44-52 weeks post-enrolment). DXA measurements outside this window will not be accepted for primary analysis. Similarly, 96-week outcome will be eligible if they are measured between 92- and 100-weeks post-enrolment.

## Measures

### Baseline measures

The following measures are recorded at baseline: TBLH and lumbar spine DXA scan, anthropometry (standing height, sitting height, weight), grip strength, long jump length, dietary assessment, IPAQ physical activity assessment, record of current symptoms, clinical and HIV history, history of fractures (Table 1). Sociodemographic variables recorded at baseline include age, sex, education level, parents, education level, household income and household asset list for calculation of socioeconomic status. See Table 2 for a list of the baseline information that will be reported in the main trial paper.

Blood samples are also taken at baseline, and rectal swabs are taken from 120 participants per country. These are for immunologic and microbiome studies which are not described in this SAP.

### Primary outcome measures

The primary outcome is mean Total Body Less-Head Bone Mineral Density (TBLH-BMD) Z-score 48 weeks after initiation of trial drug. This outcome is measured with a whole-body scan using Dual-energy X-ray Absorptiometry (DXA). The DXA machines used in the trial are Hologic DISCOVERY (Zambia) and Lunar iDXA (Zimbabwe).

TBLH-BMD and LS-BMAD (a secondary outcome) are measured in units of grams/cm^2^. They are converted to a Z-score adjusting for sex and age, using L (skewness), M (median) and S (coefficient of variation) values from a UK reference population of 3598 children and young adults [2], using the following equation where y = the measured value:

$$Z={((\frac{y}{M})}^{L}-1)/(L*S)$$

Age (in years to 0.5 years) is derived from date of birth. Values of L, M and S are shown in Appendix 1.

The mean TBLH-BMD Z-score will be compared between trial arms 48 weeks after initiation of treatment.

### Secondary outcome measures

- Lumbar Spine Bone Mineral Apparent Density (LS-BMAD) Z-score at 48 weeks, measured using a DXA lumbar spine scan.
- LS-BMAD and TBLH-BMD Z-scores at 96 weeks, measured in the same way as described above for the 48-week outcomes
- Lean muscle mass (kg) for height squared at 48 and 96 weeks, measured using a DXA body scan.
- Grip strength (kg) at 48 and 96 weeks, measured using a dynamometer. The measurement will be carried out three times with each hand, and the highest value of the six will be taken as the outcome measure.
- Number of respiratory tract infections after 48 and 96 weeks, measured using self-report of respiratory tract infections at each study visit. A respiratory tract infection will be determined on the basis of symptoms reported, as described in Appendix 1.

### Bone mechanistic sub study measures

Bone turnover markers

Bone turnover markers measured in this study are serum CTX as a marker of bone resorption and P1NP as a marker of formation. The P1NP:CTX ratio indicates net bone growth (formation relative to resorption). Plasma samples will be taken at 48 and 96 weeks for extraction of Mean P1NP:CTX ratio will be reported by trial arm at 48 and 96 weeks. We will measure P1NP and CTX by electro chemiluminescence immunoassays on plasma samples at baseline, 48- and 96-weeks post-enrolment.

Vitamin D pathway metabolites

The Vitamin D pathway metabolites are 25(OH)D, 1,25(OH)2D, 24,25(OH)2D and PTH.

We will measure 25(OH)D, 1,25(OH)2D, 24,25(OH)2D by Tandem MS and intact PTH by electro chemiluminescence immunoassays on plasma samples at baseline, 48- and 96-weeks post-enrolment.

For each pathway metabolite the mean will be reported by trial arm at 48 and 96 weeks. If the variable is not normally distributed it will be log transformed.

Tibial bone architecture

We will measure cross-sectional area, cortical area, cortical thickness, periosteal circumference, endosteal circumference, cortical volumetric bone mineral density (vBMD), vBMD at the 4% site and trabecular vBMD, at 48 and 96 weeks after initiation of trial drug using pQCT scans and report these outcomes by arm. Means will be reported if they are normally distributed, otherwise a transformation will be used.

Predicted tibial bone strength

Buckling ratio, cross-sectional moment of inertia and strength strain index of the tibia will be measured using pQCT tibial scans 48 and 96 weeks after initiation of trial drug and reported by trial arm.

Lower limb muscle and sub-cutaneous fat volume

Muscle cross-sectional area and muscle density will be measured using pQCT tibial scans 48 and 96 weeks after initiation of trial drug and reported by trial arm.

### Moderators of treatment effects

Bone age

An iDXA image of the non-dominant hand and wrist will be taken and used to quantify bone age using the Greulich and Pyle (G&P) atlas in Zimbabwe. Bone age will be compared to chronological age.

Pubertal stage

Tanner staging will be measured to assess pubertal stage (Appendix 1).

### Safety measures

At 12 weeks, blood samples will be taken and tested for calcium, phosphate and creatinine. Calcium testing will be repeated at 24 weeks. Results outside normal ranges will be confirmed by repeat laboratory measurement. Creatinine clearance will be calculated using the Schwartz formula. The number and proportion of participants with hypocalcaemia and hypercalcaemia at 12 and 24 weeks, high phosphate at 12 and 24 weeks, and high creatinine clearance at 12 weeks will be reported.

Normal calcium reference ranges are

- Zambia: 8.5 – 10.5 mg/dl
- Zimbabwe: 2.05 – 2.7 mmol/l

## Sample size considerations

A sample size of 840 participants with 15% loss to follow-up will have 80% power to detect a 0.25 effect size (standardised mean difference) for the TBLH-BMD Z-score difference between intervention and placebo arms, and 90% power to detect a 0.29 effect size.

# Data analysis plan – data description

## Recruitment and representativeness of participants

The flow of participants through each stage of screening, consent, enrolment into the study and follow-up to study exit will be illustrated using a diagram as per CONSORT guidelines (Figure 1). This will include the number of participants by arm who withdrew, were lost to follow=up, were excluded from analysis, and the number analysed. The primary outcome is measured at 48 weeks. Some secondary outcomes are measured at 96 weeks. The reasons for ineligibility or non-enrolment at screening will be shown.

## Baseline comparability of randomised groups

Characteristics of participants measured at baseline will be described by trial arm according to the analysis principles already described (Table 2). The following baseline variables will be described by arm: sex, age, school registration, orphanhood, socioeconomic status quintile, dietary calcium, level of physical activity, time since HIV diagnosis and age at diagnosis, age at ART initiation and duration on ART, viral suppression, CD4 count, ART line and regimen, cotrimoxazole use, height-for-age z-score, BMI-for-age Z-score, sitting height-for-age z-score, grip strength, pubertal stage, and TBLH-BMD Z-score and LS-BMAD Z-score. Standardised differences will be calculated for continuous variables. Statistical tests for imbalance between trial arms will not be carried out because any difference is due to chance by definition if the randomisation has been conducted correctly.

For skewed continuous variables, either geometric mean or the median and inter-quartile range (IQR) will be presented, and for categorical variables the number, total, and percentage in each category.

## Completion of treatment and adherence

The number proportion of participants who do not complete 48 weeks of treatment will be reported by arm, with the reasons for non-completion.

Adherence will be defined using pill count and number of returned tablets. For Vitamin D adherence the number of pills given to the participant over all study visits, minus the number returned, will be calculated as a percentage out of 48 (full adherence of 1 dose per week). For calcium adherence the number of pills given out over all study visits minus the number returned will be calculated as a percentage of 336 (1 dose per day for 48 weeks). If a participant ended treatment early this will be adjusted. For example, if a participant’s treatment was stopped at 24 weeks their Vitamin D adherence will be calculated as a proportion of 24, not 48.

A binary adherence variable will be created, defined as adherent (≥90% adherence to both Vitamin D and calcium) and non-adherent (<90% adherence to either Vitamin D, calcium or both).

In the intervention group, adherence will be validated against measured rise in 25(OH)D from blood samples.

## Loss to follow-up and other missing data

The number and proportions of participants missing each primary and secondary outcome variable will be summarised by trial arm and overall at each assessment time point (12, 24, 36, 48, 60, 72 and 96 weeks post-enrolment). The numbers withdrawing from the trial (i.e. actively state they are unwilling to provide any further research data) will be summarised by their reasons for withdrawal at each assessment time point by trial arm and overall.

## Adverse event reporting

Adverse events (AE), and serious adverse events (SAE) will be summarised as number of events and number of people having events by trial arm and overall. SAEs will be identified as study-related or non-study-related.

## Assessment of repeatability of outcomes

DXA, pQCT and grip strength

Measurements will each be repeated after 2 weeks in a group of 60 participants per country (120 in total). The coefficient of variation will be calculated, and a Bland-Altman plot will be drawn to determine repeatability and agreement (respectively) of measurements. A coefficient of variation of <2.5% will be considered acceptable repeatability.

Bone age

For intra-observer reliability, 10% of the iDXA images will be randomly selected and re-scored by the same trained operator after one week. For inter-observer reliability, a different set of 10% of the iDXA images will be re-scored by a second expert.

## Descriptive statistics for outcome measures

The outcome variables TBLH-BMD Z-score, LS-BMAD Z-score, lean muscle mass and upper limb grip strength are continuous variable and are expected to be normally distributed. The mean and standard deviation will be presented at baseline, 48 and 96 weeks.

For event data (number of respiratory tract infections), the number of events, person-time at risk and rate will be presented.

# Data analysis plan – inferential analysis

## Analysis principles

Primary and secondary outcomes from all randomised participants will be analysed based on a modified intention to treat (mITT) principle (4) adjusting for trial site as a binary covariate. That is, participants will be analysed in the group to which they were randomised regardless of whether they received the assigned treatment. Participants who withdraw consent retrospectively for the use of their data will not contribute to analyses. The trial statistician will remain blinded throughout analysis.

It is expected that due to the randomisation process, there will be few differences in baseline characteristics between study arms. To increase statistical power, baseline values of continuous outcomes will be adjusted for as analysis of covariance.

## Main analysis of treatment difference

### Analysis of primary outcome

The primary outcome is a continuous variable and is expected to be normally distributed. Mean values and standard deviations of TBLH-BMD Z-score for each trial arm will be reported. Linear regression will be used to compare trial arms, to estimate the mean difference and corresponding 95% confidence interval (CI), adjusting for site and baseline value of the measure as a continuous covariate. Significance tests will be two-sided with 5% level of significance and reported using overall Wald p-values. Model assumptions will be checked using standard methods.

### Analysis of secondary outcomes

Secondary outcomes, compared between trial arms, are:

a) Mean TBLH-BMD Z-score 96 weeks after treatment initiation of trial drug

b) Mean LS-BMAD Z-score 48 and 96 weeks after treatment initiation of trial drug

c) Mean lean muscle mass for height squared 48 and 96 weeks after initiation of trial drug

d) Mean upper limb grip strength 48 and 96 weeks after initiation of trial drug

These are all continuous outcomes and are expected to be normally distributed. For 48-week outcomes, linear regression will be used to compare trial arms, adjusting for site and baseline value of the measure.

The mean difference at 96 weeks (and associated 95% CI) in outcomes by trial arm, and a standardised effect size, will be estimated using a linear mixed effects model with maximum likelihood methods, with the 48-week and 96-week measures as dependent variables; a random intercept at the participant level; and baseline measure, site, trial arm, time, and trial arm by time interaction terms as independent variables.

e) Number of respiratory tract infections at 48 and 96 weeks

Number of respiratory tract infections will be reported by trial arm. The rate in person-years will be calculated based on the total time under observation. Poisson regression with robust standard errors (to allow for the lack of independence among multiple events on the same participant) will be used to compare trial arms, to estimate the incidence rate ratio and corresponding 95% CI, adjusting for site. Follow-up time will stop one week after the last dose was consumed unless a participant is lost-to-follow-up, withdraws from the study or dies. Censoring will occur at last clinic visit, date of notification of withdrawal, or reported date of death in that case.

## Statistical considerations

### TBLH-BMD and adjustment for height

In clinical practice TBLH-BMD Z-scores in children and adolescents must be adjusted for height, as TBLH-BMD is an aerial measure dependent on bone size that changes with linear growth. We have made the decision not to adjust for height in this trial because height distribution is expected to be similar between participants in the intervention and control arms, and therefore height adjustment is not needed for the purpose of the trial which is to compare the two arms. TBLH-BMD Z-score and LS-BMAD Z-score are not intended for clinical use. Should trial data be used for secondary observational analyses consideration of height adjustment will be needed for clinical inference.

### Time points

Follow-up visit should be completed no more than 4 weeks before or after the scheduled times, defined from the date of enrolment. We will report the proportion of visits which are outside this window period. Primary and secondary outcome outside the window period will be excluded. If a participant is late for a visit, the next appointment will be made to the original schedule.

### Stratification

Randomisation is stratified by site, and therefore site (country) will be included as a covariate in all analysis.

### Missing baseline data

The amount (number, percentage) of data that is missing will be reported for each of the key outcome variables and covariates. Baseline characteristics of participants with and without missing data will be compared. T-tests or linear regression will be used for normally distributed continuous covariates comparing the mean values of those with and without missing data, Mann Whitney nonparametric test for continuous skewed covariates, and chi squared tests and percentages for categorical covariates. An assessment for effect modification of missingness by trial arm will be made by incorporating an interaction term between the baseline characteristic and trial arm.

### Missing outcome data

We will use the 48-week outcome from the maximum likelihood mixed models of 48- and 96-week TBLH-BMD Z-score to adjust for missing data in the primary outcome. We will not conduct multiple imputation for secondary outcomes.

### Per protocol analysis

Per protocol adherence to the study drug will be defined using percentage increase in 25(OH)D level from baseline to 48 weeks among intervention arm participants.

All control arm participants will be defined as per protocol adherent. In the intervention arm, a cut point for percentage increase in 25(OH)D compared to baseline will be predetermined. Participants above this cut point will be defined as per protocol adherent and those below the cut point will be dropped from per protocol analysis. The percentage increase cut point will be decided following examination of baseline 25(OH)D levels, and before unblinding. After unblinding, the continuous vitamin D adherence percentile will be validated against 48-week plasma 25(OH)D levels for participants in the intervention arm.

### Method for handling multiple comparisons

We do not plan to adjust for multiple comparisons.

## Sensitivity analysis

No sensitivity analysis is planned.

## Planned subgroup analysis

Pre-specified subgroup analyses on the primary outcome (TBLH-BMD at 48 weeks) will be carried out by

- Trial site (Zambia, Zimbabwe)
- 25(OH)D level at enrolment (subgroups to be determined after examination of 25(OH)D distribution at enrolment)
- Age group (age at enrolment in years: 11-13, 14-16, 17-19)
- Sex (Male, Female)
- Pubertal stage (Tanner stage at enrolment: 1-3, 4-5)
- Age at peak height velocity (PHV) in year of intervention (before, during or after PHV) (Appendix 2)

Effect modification will be examined by incorporating an interaction term between subgroups and trial arm. In Zimbabwe only, subgroup analysis on TBLH-BMD at 48 weeks will be carried out by bone age at enrolment.

## Interim analysis

No interim analysis is planned.

## Process of unblinding

A final cleaned dataset will be produced after the last follow-up visit has been completed. Distributional assumptions will be tested, exclusions will be defined, and the analysis plan will be finalised. A blinded analysis will then be conducted by the trial statistician with the allocated arms labelled A and B as provided by the independent statistician. The blinded results will be discussed and interpreted at an investigator’s meeting. An envelope containing the treatment allocation will be opened after this discussion at the investigator’s meeting.

# Software

ODK is used for data management, hosted on a server at the London School of Hygiene & Tropical Medicine and managed by the trial data manager. R will be used for the data description and the main inferential analysis.

# Schedule of assessments and measures

This is shown in Table 1.

# Figure 1: CONSORT Flow Diagram VITALITY trial

Allocated to Vit D/calcium (n= )

¨ Received allocated drugs (n= )

¨ Did not receive allocated drugs (reasons) (n= )

Allocated to placebo (n= )

¨ Received allocated placebo (n= )

¨ Did not receive allocated placebo (reasons) (n= )

**Enrolment**

Lost to follow-up (give reasons) (n= )

Discontinued Vit D/calcium (reasons) (n= )

Lost to follow-up (reasons) (n= )

Discontinued placebo (reasons) (n= )

**Follow-Up**

Analysed at 48 weeks (n= )
¨ Excluded from analysis (reasons) (n= )

Analysed at 96 weeks (n= )
¨ Excluded from analysis (reasons) (n= )

Analysed at 48 weeks (n= )
¨ Excluded from analysis (reasons) (n= )

 Analysed at 96 weeks (n= )
¨ Excluded from analysis (reasons) (n= )

**Analysis**

Assessed for eligibility (n= )

Excluded (n= )

¨  Not meeting non-clinical inclusion criteria (n= )

¨  Not meeting clinical inclusion criteria (n= )

¨  Declined to participate (n= )

¨  Other reasons (n= )

Randomised (n= )

**Allocation**

## Table 1: schedule of assessments

| **Procedures** |  | **Weeks since enrolment** | | | | | | | | |
| --- | --- | --- | --- | --- | --- | --- | --- | --- | --- | --- |
|  | **0** | **2** | **12** | **24** | **36** | **48** | **60*** | **72** | **84*** | **96** |
| Demographics, clinical & HIV history^a^ | **✓** |  |  |  |  |  |  |  |  |  |
| Current symptoms / Adverse events | **✓** | **✓** | **✓** | **✓** | **✓** | **✓** | **✓** | **✓** | **✓** | **✓** |
| Dietary assessment & physical activity | **✓** |  |  |  |  | **✓** |  |  |  | **✓** |
| Anthropometry^b^ | **✓** |  | **✓** | **✓** | **✓** | **✓** | **✓** | **✓** | **✓** | **✓** |
| pGALS | **✓** |  |  |  |  | **✓** |  | **✓** |  | **✓** |
| Grip strength | **✓** |  |  |  |  | **✓** |  |  |  | **✓** |
| Pubertal Staging | **✓** |  |  |  |  | **✓** |  |  |  | **✓** |
| TBLH and Lumbar spine DXA scan | **✓** |  |  |  |  | **✓** |  |  |  | **✓** |
| pQCT scan (Zimbabwe only) | **✓** |  |  |  |  | **✓** |  |  |  | **✓** |
| Hand x-ray/iDXA (Zimbabwe only) | **✓** |  |  |  |  | **✓** |  |  |  | **✓** |
| Supply Trial drug | **✓** | **✓** | **✓** | **✓** | **✓** |  |  |  |  |  |
| Measurement of adherence to trial drug |  | **✓** | **✓** | **✓** | **✓** | **✓** |  |  |  |  |
| CD4 count and viral load | **✓** |  |  |  |  | **✓** |  |  |  | **✓** |
| Vitamin D pathway metabolites^c^; Bone turnover markers^d^; iPTH | **✓** |  |  |  |  | **✓** |  |  |  | **✓** |
| Safety bloods^e^ |  |  | **✓** | **✓** |  |  |  |  |  |  |
| Blood samples for immunology studies | **✓** |  |  |  |  | **✓** |  |  |  | **✓** |
| Rectal swab for microbiome studies^f^ | **✓** |  |  |  |  | **✓** |  |  |  | **✓** |
| Full blood count (Zambia only) | **✓** |  |  |  |  |  |  |  |  |  |
| BIA & ADP (Zambia only) | **✓** |  |  |  |  | **✓** |  |  |  | **✓** |

*^a^Age at diagnosis, WHO disease stage, nadir CD4 count, ART regimen and duration of treatment; ^b^Standing and sitting height and weight, (plus calf waist and hip circumference and skin fold thickness (triceps, subscapular and suprailiac measurements in Zambia only)  ^c^25OHD, 1,25(OH)_2_D, 24,25(OH)_2_D); ^d^P1NP, CTX; ^e^calcium, phosphate (12 and 24 weeks), creatinine (12 weeks only); ^f^first 120 participants recruited only; *Brief visit: to maximise retention*

## Table 2: Baseline characteristics

| **Baseline characteristics** | Vitamin D and calcium arm | Placebo arm |
| --- | --- | --- |
| **Demographic** |  |  |
| Sex, female, n (%) |  |  |
| Age, median, y |  |  |
| Registered for school attendance currently, n (%) |  |  |
| Orphanhood |  |  |
| SES quintile |  |  |
| Dietary calcium, median |  |  |
| Physical activity (MET mins/week) |  |  |
| **HIV** |  |  |
| Time since diagnosis, median, y |  |  |
| Age at diagnosis, median, y |  |  |
| Duration on ART, median, y |  |  |
| Age at ART start, median, 7 |  |  |
| VL<1000 copies/ml, n (%) |  |  |
| CD4, median, cells/mm^3^ (IQR) |  |  |
| Second line ART, n (%) |  |  |
| ART Regimen, n (%) |  |  |
| DTG |  |  |
| EFV/NVP |  |  |
| ATV/LPV |  |  |
| Other |  |  |
| Taking cotrimoxazole prophylaxis, n (%) |  |  |
| **Anthropometric** |  |  |
| TBLH-BMD Z-score, mean (SD) |  |  |
| LS-BMAD Z-score, mean (SD) |  |  |
| Height-for-age z-score, mean (SD) |  |  |
| Height-for-age z-score <-2, n (%) |  |  |
| BMI-for-age z-score, mean (SD) |  |  |
| BMI-for-age z-score <-2, n (%) |  |  |
| Sitting-height-for-age z-score, mean (SD) |  |  |
| Grip strength, mean (SD) |  |  |
| Tanner stage 4-5, n (%) |  |  |

## Table 3: Effect of the intervention

| **Outcome** | **Vitamin D and calcium arm** | **Placebo arm** | **Adjusted Mean Difference (95% CI)** |
| --- | --- | --- | --- |
|  | **Mean (SD)** | **Mean (SD)** |  |
| **TBLH-BMD Z-score at 48 weeks, mean (SD)** |  |  |  |
| **TBLH-BMD Z-score at 96 weeks, mean (SD)** |  |  |  |
| **LS-BMAD Z-score at 48 weeks, mean (SD)** |  |  |  |
| **LS-BMAD Z-score at 96 weeks, mean (SD)** |  |  |  |
| **Lean muscle mass** for height squared **at 48 weeks, mean (SD)** |  |  |  |
| **Lean muscle mass** for height squared **at 96 weeks, mean (SD)** |  |  |  |
| **Grip strength (kg) at 48 weeks, mean (SD)** |  |  |  |
| **Grip strength (kg) at 96 weeks, mean (SD)** |  |  |  |

## Table 4: Effect of the intervention

|  | **Vitamin D and calcium arm** | | | **Placebo arm** | | |  |
| --- | --- | --- | --- | --- | --- | --- | --- |
|  | **Events** | **Person years** | **Rate/100py (95% CI)** | **Events** | **Person years** | **Rate/100py (95% CI)** | **Adjusted Rate Ratio (95% CI)** |
| **Respiratory tract infections at 48 weeks** |  |  |  |  |  |  |  |
| **Respiratory tract infections at 96 weeks** |  |  |  |  |  |  |  |

# References

1. Schulz KF, Altman DG, CONSORT Group: **CONSORT 2010 statement: updated guideliens for reporting parallel group randomised trials**. *BMJ* 2010, **340**:c332.

2. Crabtree N, Shaw NJ, N.J. B, Adams JE, Mughal MZ, Aundel P, Fewtrell MS, Ahmed F, Treadgold LA, Hogler W *et al*: **Amalgamated Reference Data for Size-Adjusted Bone Densitometry Measurements in 3598 Children and Young Adults – the Alphabet Study**. *J Bone Miner Res* 2017, **32**(1):172-180.

3. Rukuni R, Rehman AM, Mukwasi-Kahari C, Madanhire T, Kowo-Nyakoko F, McHugh G, Filteau S, Chipanga J, Simms V, Mujuru H *et al*: **Effect of HIV infection on growth and bone density in peripubertal children in the era of antiretroviral therapy: a cross-sectional study in Zimbabwe**. *Lancet Child Adolesc Health* 2021, **5**(8):569-581.

# Appendix 1: scoring

TBLH BMD

The L, M and S values for the Hologic and iDXA by age and sex are shown below [2].

|  | **Hologic** | | | | | | **iDXA** | | | | | |
| --- | --- | --- | --- | --- | --- | --- | --- | --- | --- | --- | --- | --- |
|  | **Girls** | | | **Boys** | | | **Girls** | | | **Boys** | | |
| **Age (y)** | **L** | **M** | **S** | **L** | **M** | **S** | **L** | **M** | **S** | **L** | **M** | **S** |
| **11** | 1.00 | 0.756 | 0.08 | 1.00 | 0.751 | 0.08 | 0.08 | 0.741 | 0.10 | 0.29 | 0.737 | 0.10 |
| **11.5** | 1.00 | 0.778 | 0.08 | 1.00 | 0.768 | 0.08 | 0.08 | 0.768 | 0.10 | 0.29 | 0.759 | 0.10 |
| **12** | 1.00 | 0.800 | 0.08 | 1.00 | 0.786 | 0.08 | 0.08 | 0.795 | 0.10 | 0.29 | 0.783 | 0.10 |
| **12.5** | 1.00 | 0.822 | 0.08 | 1.00 | 0.807 | 0.08 | 0.08 | 0.824 | 0.10 | 0.29 | 0.810 | 0.10 |
| **13** | 1.00 | 0.842 | 0.08 | 1.00 | 0.833 | 0.08 | 0.08 | 0.853 | 0.10 | 0.29 | 0.841 | 0.10 |
| **13.5** | 1.00 | 0.860 | 0.08 | 1.00 | 0.863 | 0.08 | 0.08 | 0.881 | 0.10 | 0.29 | 0.875 | 0.10 |
| **14** | 1.00 | 0.876 | 0.08 | 1.00 | 0.896 | 0.08 | 0.08 | 0.905 | 0.10 | 0.29 | 0.911 | 0.11 |
| **14.5** | 1.00 | 0.890 | 0.08 | 1.00 | 0.930 | 0.08 | 0.08 | 0.926 | 0.10 | 0.29 | 0.946 | 0.11 |
| **15** | 1.00 | 0.902 | 0.08 | 1.00 | 0.961 | 0.08 | 0.08 | 0.943 | 0.09 | 0.29 | 0.980 | 0.11 |
| **15.5** | 1.00 | 0.913 | 0.08 | 1.00 | 0.990 | 0.08 | 0.08 | 0.956 | 0.09 | 0.29 | 1.010 | 0.11 |
| **16** | 1.00 | 0.921 | 0.08 | 1.00 | 1.015 | 0.08 | 0.08 | 0.967 | 0.09 | 0.29 | 1.037 | 0.11 |
| **16.5** | 1.00 | 0.929 | 0.08 | 1.00 | 1.036 | 0.08 | 0.08 | 0.975 | 0.09 | 0.29 | 1.061 | 0.12 |
| **17** | 1.00 | 0.937 | 0.08 | 1.00 | 1.053 | 0.08 | 0.08 | 0.980 | 0.09 | 0.29 | 1.082 | 0.12 |
| **17.5** | 1.00 | 0.943 | 0.08 | 1.00 | 1.068 | 0.08 | 0.08 | 0.983 | 0.09 | 0.29 | 1.101 | 0.12 |
| **18** | 1.00 | 0.950 | 0.08 | 1.00 | 1.080 | 0.08 | 0.08 | 0.985 | 0.09 | 0.29 | 1.118 | 0.12 |
| **18.5** | 1.00 | 0.956 | 0.08 | 1.00 | 1.091 | 0.08 | 0.08 | 0.986 | 0.09 | 0.29 | 1.134 | 0.12 |
| **19** | 1.00 | 0.962 | 0.08 | 1.00 | 1.102 | 0.08 | 0.08 | 0.986 | 0.09 | 0.29 | 1.149 | 0.12 |
| **19.5** | 1.00 | 0.969 | 0.08 | 1.00 | 1.112 | 0.08 | 0.08 | 0.987 | 0.09 | 0.29 | 1.163 | 0.12 |
| **20** | 1.00 | 0.975 | 0.08 | 1.00 | 1.123 | 0.08 | 0.08 | 0.987 | 0.09 | 0.29 | 1.177 | 0.12 |

LS-BMAD

The L, M and S values for the Hologic and iDXA by age and sex are shown below [2].

|  | **Hologic** | | | | | | **iDXA** | | | | | |
| --- | --- | --- | --- | --- | --- | --- | --- | --- | --- | --- | --- | --- |
|  | **Girls** | | | **Boys** | | | **Girls** | | | **Boys** | | |
| **Age (y)** | **L** | **M** | **S** | **L** | **M** | **S** | **L** | **M** | **S** | **L** | **M** | **S** |
| **11** | 1.00 | 0.206 | 0.11 | 1.00 | 0.187 | 0.13 | -0.01 | 0.273 | 0.11 | 1.00 | 0.256 | 0.11 |
| **11.5** | 1.00 | 0.212 | 0.11 | 1.00 | 0.190 | 0.13 | -0.01 | 0.276 | 0.11 | 1.00 | 0.256 | 0.11 |
| **12** | 1.00 | 0.217 | 0.11 | 1.00 | 0.193 | 0.13 | -0.01 | 0.281 | 0.11 | 1.00 | 0.257 | 0.11 |
| **12.5** | 1.00 | 0.223 | 0.11 | 1.00 | 0.197 | 0.12 | -0.01 | 0.287 | 0.11 | 1.00 | 0.258 | 0.11 |
| **13** | 1.00 | 0.229 | 0.11 | 1.00 | 0.203 | 0.12 | -0.01 | 0.295 | 0.11 | 1.00 | 0.259 | 0.11 |
| **13.5** | 1.00 | 0.234 | 0.11 | 1.00 | 0.209 | 0.12 | -0.01 | 0.303 | 0.11 | 1.00 | 0.262 | 0.11 |
| **14** | 1.00 | 0.238 | 0.11 | 1.00 | 0.215 | 0.11 | -0.01 | 0.311 | 0.11 | 1.00 | 0.267 | 0.11 |
| **14.5** | 1.00 | 0.242 | 0.11 | 1.00 | 0.222 | 0.11 | -0.01 | 0.317 | 0.11 | 1.00 | 0.274 | 0.11 |
| **15** | 1.00 | 0.245 | 0.11 | 1.00 | 0.228 | 0.10 | -0.01 | 0.320 | 0.11 | 1.00 | 0.281 | 0.11 |
| **15.5** | 1.00 | 0.248 | 0.11 | 1.00 | 0.234 | 0.10 | -0.01 | 0.322 | 0.11 | 1.00 | 0.286 | 0.11 |
| **16** | 1.00 | 0.250 | 0.11 | 1.00 | 0.238 | 0.10 | -0.01 | 0.323 | 0.11 | 1.00 | 0.291 | 0.11 |
| **16.5** | 1.00 | 0.252 | 0.11 | 1.00 | 0.242 | 0.09 | -0.01 | 0.324 | 0.11 | 1.00 | 0.295 | 0.11 |
| **17** | 1.00 | 0.254 | 0.11 | 1.00 | 0.244 | 0.09 | -0.01 | 0.325 | 0.11 | 1.00 | 0.297 | 0.11 |
| **17.5** | 1.00 | 0.255 | 0.11 | 1.00 | 0.246 | 0.09 | -0.01 | 0.325 | 0.11 | 1.00 | 0.299 | 0.11 |
| **18** | 1.00 | 0.256 | 0.11 | 1.00 | 0.248 | 0.09 | -0.01 | 0.326 | 0.11 | 1.00 | 0.300 | 0.11 |
| **18.5** | 1.00 | 0.258 | 0.11 | 1.00 | 0.249 | 0.09 | -0.01 | 0.326 | 0.11 | 1.00 | 0.301 | 0.11 |
| **19** | 1.00 | 0.259 | 0.11 | 1.00 | 0.250 | 0.09 | -0.01 | 0.326 | 0.11 | 1.00 | 0.301 | 0.11 |
| **19.5** | 1.00 | 0.260 | 0.11 | 1.00 | 0.250 | 0.08 | -0.01 | 0.326 | 0.11 | 1.00 | 0.302 | 0.11 |
| **20** | 1.00 | 0.261 | 0.11 | 1.00 | 0.251 | 0.08 | -0.01 | 0.326 | 0.11 | 1.00 | 0.302 | 0.11 |

Respiratory tract infection

A respiratory tract infection is defined as experiencing

- any 2 or more of the following symptoms: fever, dry cough, runny stuffy nose, sore throat, sneeze, productive cough, difficulty breathing, rapid heartbeat, chest pain/discomfort.
- With none of the following allergy symptoms: watery eyes, itchy nose or eyes, clear nasal discharge

These symptoms will be recorded at every study visit.

Pubertal stage

Tanner scoring will be used to assess pubertal stage. For females, pubic hair development and breast development will be scored from 1 (prepubertal) to 5. For males, pubic hair and testes development will be scored. Tanner stage will be defined as breast development score for females and testes stage for males. Pubertal stage will be categorised as: Tanner score 1-3=early puberty, 4-5=late puberty.

Pubertal delay will be defined as any participant who, at any time point:

- Has not started menstruation at age ≥ 16 years (female)
- Is at Tanner stage 1 for breast development when aged ≥ 13 years (female)
- Is at Tanner stage 1 for testes development when aged ≥ 14 years (male)

Socioeconomic status (SES)

Data will be collected on whether the participant’s household has the following: electricity supply, a refrigerator, a bicycle, a motorbike, a car, a television, a radio, a mobile phone, a computer. Data will also be collected on main water source, type of toilet, ownership of the home (own, rent the main dwelling, rent part of the dwelling) and household income. Factor analysis will be applied to these data to create a variable for socioeconomic status, stratified by country. This continuous variable will be divided into quintiles from poorest to least poor.

International Physical Activity Questionnaire (IPAQ-short form)

The frequency and duration of vigorous and moderate physical activity, and walking, will be used to calculate physical activity as multiples of the resting metabolic rate (MET) in MET-minutes.

Standing height

Standing height will be measured 3 times using a wall-mounted stadiometer. If the three measurements are within 2cm of each other the mean will be used as the outcome. if the range is great, the measurements will be examined, any outlier values will be dropped, and the mean of the remaining values calculated. Height for age z-score will be calculated using the UK reference range.

Sitting height

Standing height will be measured 3 times using a wall-mounted stadiometer while seated on a box or stool. The height of the box is recorded separately. Mean height will be determined using the same method as for standing height. Sitting height for age z-score will be calculated using the UK reference range.

Weight

Weight will be measured 3 times using an electronic scale. The mean of the 3 values will be used as the outcome. BMI will be calculated as weight^2^/standing height. Z-scores for weight for age and BMI for age will be calculated using the UK reference ranges.

Grip strength

Hand grip strength will be measured using a Jamar hydraulic hand-held dynamometer (Patterson Medical, UK) to the nearest 0.1kg. Participants will be seated with the shoulder at 0° to 10°, the elbow at 90° of flexion and the forearm positioned neutrally. Three measurements will be taken from each hand in alternation and the highest measurement chosen.

Long jump

The standing long jump distance will be taken from the best of three correctly performed attempts to the nearest 0.1 cm, measuring the distance from the take-off line to the heel.

HIV viral load

Viral load is measured in units of copies/mL. A binary variable for viral suppression will be created, defined as suppressed (<1000 copies/mL) and unsuppressed (≥1000 copies.mL).

Dietary calcium

Dietary calcium will be measured using a food frequency scale. Data will be collected on the number of times per week (never, less than once 1-2 times, 3-5 times, almost every day) participants consume each of the following foods: legumes, dairy products, meat, eggs, fish and fat. Standardised quantities of the amount of calcium per daily serving in each of these foods will be used to estimate dietary calcium intake.

Vitamin D exposure

Data will be collected on how much time participant spend out of doors during daylight hours per day (none, less than 1 hour, 1-2 hours, >2 hours) and how much of their skin is usually exposed (just face and hands; face, hands, arms or legs; often no shirt as well as face, hands, arms or legs). Vitamin D dietary intake will be calculated using standard quantities of Vitamin D found in the 6 food groups listed above for calcium.

# Appendix 2: Peak height velocity

Peak height velocity (PHV) is defined as the period during which an adolescent experiences the fastest growth in height. By measuring height at frequent intervals throughout puberty and calculating the speed of growth, PHV can be determined. In the VITALITY trial participants will have their height recorded at 12-week intervals for 96 weeks. Height velocity will be calculated in cm/year. If PHV occurs during the period of observation (i.e. if height velocity reaches a maximum and slows down), the age of PHV can be determined. Adolescents who are observed from Tanner stage measurements to be pre-pubertal and who do not have an observed PHV will be categorised as pre-PHV. Those who are fully developed according to Tanner stage and who do not have an observed PHV will be categorised as post-PHV. In all remaining cases information on PHV will be coded as missing.
